# Barriers and facilitators to postoperative pain management in Rwanda from the perspective of health care providers: A contextualization of the theory of planned behavior

**DOI:** 10.1080/24740527.2018.1451251

**Published:** 2018-04-12

**Authors:** Gaston Nyirigira, Rosemary A. Wilson, Elizabeth G. VanDenKerkhof, David H. Goldstein, Theogene Twagirumugabe, Ryan Mahaffey, Joel Parlow, Ana P. Johnson

**Affiliations:** a Anaesthesia, Critical Care and Pain, Butare University Teaching Hospital, Butare, Rwanda; b School of Nursing, Queen’s University, Kingston, Ontario, Canada; c Department of Anesthesiology and Perioperative Medicine, Queen’s University, Kingston Health Sciences Centre, Kingston, Ontario, Canada; d Department of Anesthesiology, Brockville General Hospital, Brockville, Ontario, Canada; e College of Medicine and Health Sciences, University of Rwanda, Kigali, Rwanda; f Department of Public Health Sciences, Queen’s University, Kingston, Ontario, Canada

**Keywords:** Acute pain, pain assessment, pain treatment, knowledge translation

## Abstract

**Aims:**

Identify opportunities to improve knowledge translation for post-operative pain management in Rwanda by exploring clinician and environmental factors affecting this practice.

**Methods:**

The theory of planned behavior (TPB) guided development of a questionnaire to measure intent to assess and treat postoperative pain. Focus groups and individual interviews were used to contextualize the final questionnaire and generate questions related to pain management practice. Health care providers from two Rwandan teaching hospitals involved in postoperative pain management completed the TPB questionnaire in May 2015. TPB subscale scores were analyzed to identify demographic and practice characteristics associated with intention to treat pain. The general linear model was used to test effect of attitudes, subjective norms, and perceived control on behavioral intent to treat pain.

**Results:**

Forty-six percent of participants (*N* = 131) had training in acute pain management, 56% used a pain protocol, and 74% used pain scales. Tramadol (78%), morphine (79%), and paracetamol (75%) were used most often to treat pain. Drug availability was the most frequently reported barrier to treating pain. Though intention to treat pain was high, only attitudes and perceived control about assessing pain were associated with intention to treat pain. The theme of fear of the adverse effects of pain medications was consistent across focus groups and interviews in both sites.

**Conclusions:**

System and knowledge barriers exist: interventions to address these barriers may lead to improved postoperative pain care. Further validation of the TPB questionnaire is required to address cultural and language factors specific to the Rwandan context.

## RÉSUMÉ

**But**: Améliorer le transfert des connaissances en matière de prise en charge de la douleur postopératoire au Rwanda en étudiant les facteurs environnementaux et les facteurs liés aux cliniciens qui affectent cette pratique.

**Méthodes**: La théorie du comportement planifié (TCP) a guidé l’élaboration d’un questionnaire visant à mesurer l’intention d’évaluer et de traiter la douleur postopératoire. Des groupes de discussion et des entrevues individuelles ont été utilisées afin de contextualiser le questionnaire final et formuler des questions portant sur les pratiques en matière de gestion de la douleur. Les prestataires de soins de deux hôpitaux universitaires rwandais impliqués dans la prise en charge de la douleur postopératoire ont répondu au questionnaire fondé sur la TCP en mai 2015. Les scores obtenus pour les sous-échelles ont été analysés afin de cerner les caractérisriques démographiques et les caractéristiques liées aux pratiques qui étaient associées à l’intention de traiter la douleur. Le modèle linéaire général a été utilisé afin de tester l’effet des attitudes, des normes subjectives et du contrôle perçu sur l’intention comportementale de traiter la douleur.

**Résultats**: Quarante-six pour cent des participants (N = 131) avaient une formation en prise en charge de la douleur aigue, 56 % utilisaient un protocole de prise en charge de la douleur et 74 % utilisaient des échelles d’évaluation de la douleur. Le tranadol (78 %), la morphine (79 %) et le paracétamol (75 %) étaient le plus souvent utilisés pour traiter la douleur. La disponibilité des médicaments était la barrière au traitement de la douleur la plus fréquemment citée. Bien que l’intention de traiter la douleur était élevée, seules les attitudes et le contrôle perçu au sujet de l’évaluation de la douleur étaient associés à l’intention de traiter la douleur. Le thème de la peur des effets indésirables des médicaments contre la douleur a été soulevé dans tous les groupes de discussions et les entrevues tenus dans les deux sites.

**Conclusions**: Il existe des barrières systémiques et des barrières liées aux connaissances : des interventions visant à lever ces barrières pourraient mener à l’amélioration des soins postopératoires. Le questionnaire fondé sur la TCP doit être validé davantage afin d’aborder les facteurs liés à la culutre et au langage spécifiques au contexte rwandais.

## Introduction

Access to safe and appropriate surgery and perioperative pain management has been designated as a basic human right.^1–3^ However, there is evidence that postoperative pain is poorly managed worldwide,^4–6^ despite advances in pain care. High rates of moderate to severe pain are reported in many settings,^7^ even where there is ready access to the latest pain therapies.^8^ In low-resource countries, where there may be limited access to the medications, technologies, and training to treat acute pain, there is wide variation in the reported prevalence of moderate to severe acute pain and the use of analgesic interventions.^9,10^ Aisuodionoe-Shadrach et al. reported that only 50% of all patients (*n* = 106) with pain in a Nigerian emergency department received analgesia, and of those receiving treatment, 81% still experienced moderate to severe residual pain.^9^ Similarly, Faponle et al. found that 69% (24 h) and 52% (48 h) of patients (*n* = 149) reported moderate to severe pain after general surgery.^10^ Faponle et al.^10^ also reported that intramuscular injection was the only route of administration offered to patients and pethidine, pentazocine, and dipyrone were the only available medications. In an Ethiopian study, 91% of patients reported moderate to severe postoperative pain in three consecutive assessments in a study conducted on 252 surgical inpatients who underwent various surgical procedures.^11^ Eighty percent of patients felt that their pain had been undertreated.^11^

Developing countries continue to face challenges with establishing and maintaining effective programs for the improvement of acute pain because of lack of expertise and resources. Barriers to evidence-informed treatment of postoperative pain occur at the levels of the patient, system, and provider.^12,13^ Patient barriers include fear of addiction and side effects of treatment^14–16^; system barriers relate to legal and regulatory obstacles based on opioid abuse and addiction^17,18^; and provider barriers relate to treating pain based on disease rather than symptoms, noncompliance with guidelines, fear of addiction, and poor knowledge, particularly about opioid pharmacology.^13,15,16^ In addition, poverty, illiteracy, and inadequate training of providers have been reported to contribute to inadequate postoperative analgesia delivery.^5^ In a study of pain practice in an urban hospital emergency room in Rwanda, nurses reported that although they felt confident in their ability to treat pain, medication availability, assessment ability, fears about medication side effects, and cultural factors affected their practice.^19^ The resultant effect was a median delay of 150 min from report to treatment or avoidance of analgesic administration in those patients who reported severe pain.

The consequences of uncontrolled acute pain are well established. Immobility and cardiovascular, respiratory, and gastrointestinal complications can have a negative effect on recovery and delay hospital discharge.^20^ Unrelieved acute pain may also increase the risk of chronic pain.^21^ Our early work, and that of others,^22^ has outlined the limited access to the drugs, technologies, and training in Rwanda to target acute pain. However, pain relief can be reliably achieved in the acute phase with the use of inexpensive medications and treatments.^23^

### Rwandan context

In 2010, 706 surgical procedures were performed per 100 000 Rwandans,^24^ and there were over 9000 road accidents, 25% of which were severe and resulted in major injuries that were fatal or required surgery.^25^ The improvement and expansion of health care in Rwanda has been the focus of a number of global health initiatives, employing collaborations with mainly academic institutions in developed countries (e.g., Rwandan Human Resources for Health,^26^ Canadian Anesthesiologists’ Society International Education Foundation^27^). Currently, institutional health care is provided through a decentralized system of five referral and 42 district hospitals, health posts, and a network of dispensaries, transfusion centers, and clinics. Surgery and perioperative care are exclusively provided at district and referral centers.^28^ There are two university teaching hospitals. University Central Hospital of Kigali (CHUK) is a 513-bed hospital located in the capital city of Kigali. University Teaching Hospital of Butare (CHUB) is a 420-bed hospital located in Butare in the southern province of Rwanda. In the operative setting, nurse anesthetists (NAs) or anesthesia technicians provide much of the anesthetic care due to the limited number of anesthesiologists in Rwanda (12 in 2010)^24^ and generally do so without the direct supervision of an anesthesiologist.

### Previous work in Rwanda

The overarching goal of our work to date has been to build clinical and research capacity in Rwanda by training individuals to develop, evaluate, and implement effective strategies for acute pain management by responding to local needs and barriers and to conduct independent research for ongoing improvement. For the past 4 years, there has been successful collaboration between Rwanda and our group in Canada on a project funded by the Canada-Africa Research Exchange Grants and Canadian Anesthesiologists’ Society International Education Foundation. This project focuses on barriers to knowledge translation in the education and implementation of acute pain care practices and facilitating the increased use of research evidence by key stakeholders using the knowledge to action (KTA) framework.^29,30^ This approach is similar to that taken by Livingston and colleagues to enhance obstetrical anesthesia in Rwanda.^31,32^

The purpose of the current study was to examine postoperative pain management practices and barriers and facilitators to providing pain care in Rwanda, with the aim of identifying opportunities to improve knowledge translation for postoperative pain management in a low resource setting.

The objectives were to (1) develop a culturally, ethically, and contextually appropriate tool for describing barriers, facilitators, and the current practice of pain assessment and treatment and (2) use the newly developed tool to describe the barriers and facilitators and the current practice of pain assessment and treatment in perioperative and posttraumatic care settings in two Rwandan referral hospitals.

## Materials and methods

This study was reviewed for ethical compliance by the Queen’s University Health Sciences and Affiliated Teaching Hospitals Research Ethics Board (ANAE-230-12) and the College of Medicine and Health Sciences at the University of Rwanda (CMHS/IRB/115/2015). Separate hospital ethics approval was obtained at both CHUK and CHUB. Written informed consent was obtained from all participants. This article adheres to the applicable EQUATOR guidelines.^33^

### Application of the KTA framework

Knowledge must be applied in an ethically guided manner, reflective of values and norms in the community, for communication and decision making about the appropriate relevant use of health care resources to be improved.^30^ The KTA framework^29,30^ guided this work and focused the effort in this phase of our program toward expanding the identification of educational needs, barriers, and facilitators to pain management practice among the whole of the interprofessional team. Within this framework, tailoring of knowledge and evidence is key to achieving a sustainable outcome. The first assessment provided us with a preliminary understanding of the problem. A description of the context and culture of the clinical environment in Rwandan referral hospitals is necessary to enable us to develop an implementation strategy for improving practice and communication among all stakeholders that could be adapted and tailored to local context and ultimately improve the quality of pain treatment provided to patients.

Consistent with the KTA framework, the intent of the first objective of the study, development of a culturally, ethically, and contextually appropriate assessment tool, would allow us to then describe the context and culture of the clinical environment (objective 2). Objective 1 involved two steps: (1) using results from our previous work^22^ to create a questionnaire based on the theory of planned behavior (TPB)^34^ and (2) refinement of the TPB questionnaire in the local context; that is, Rwanda. Objective 2 involved conducting a survey of health care providers in two Rwandan teaching hospitals using the contextualized questionnaire.

### Objective 1

In the first phase, the TPB questionnaire was developed using the findings of the previous study^22^ and consultation with Rwandan team members (GN, TT), using the process described by Ajzen.^34^ The TPB has been used extensively to identify factors that influence individuals’ intentions to enact a particular behavior or set of behaviors.^35,36^ The TPB links behavioral intent to a person’s attitude toward that behavior (Attitude), the influence of social pressure that is perceived by the person (Subjective Norms), and the person’s perception of their control over performing the behavior (Perceived Behavioral Control).^36^ The TPB has been used with some utility in African populations to examine health protective behaviors^37^ but has not been applied to pain management practice. In the second phase of objective 1, development of the tool involved an elicitation study consistent with TPB questionnaire construction process^36^ to understand the salient aspects of commonly held beliefs about pain management practice to contextualize the tool’s content. This was achieved through key informant interviews and focus groups with health care providers involved in pain management in Rwanda. Interviews and focus groups were facilitated by GN, who provided translation of general questions and subsequent discussion as necessary. The previously constructed TPB questionnaire items were reviewed for comprehension and participants were asked to suggest changes to wording, layout, and content to ensure that the final questionnaire items were written in language that was appropriate to Rwandan diction and understanding. Semistructured questions were then posed in each encounter and discussion facilitated with clarifying questions/comments added by team members (EV/RW) as appropriate. Initial questions included “What are your experiences with providing care to patients who are in pain?” “What are the medications and treatments available to assist you?” “Can you tell us about any issues/problems you have encountered providing care to patients in pain?”

The English version of the questionnaire was translated into French. It was then back-translated and revised by a Rwandan doctoral student studying at Queen’s University to ensure consistency with the Rwandan French language. Finally, it was reviewed and revised by a Rwandan physician team member (GN).

### Objective 2

Following creation of the questionnaire as described above, it was then administered to a convenience sample of physicians, anesthesiology and surgical residents, nurses, and anesthesia technicians/NAs involved in perioperative care. The sample was recruited from the postanesthetic care unit (PACU), intensive care unit (ICU), and orthopedic, general surgery, obstetric/gynecology, and pediatric surgical units over 6 days in May 2015. Though staff nurses in the operating room setting would normally not be responsible for pain management, at CHUK and CHUB the nurses rotate between the operating room and postanesthetic care and therefore all nurses in these two settings were approached to participate in the survey. Participants were given a consent and information form, which asked them to reflect on their experience treating postoperative pain. A key stakeholder was identified on each nursing unit or by specialty (e.g., surgical residents). The consent and information form and each question were reviewed by a local investigator (GN) with the key stakeholder, and further clarification in Kinyarwanda, English, and/or French was provided as needed. The questionnaire was made available in English and French. An envelope containing consent forms and questionnaires was left with key stakeholders who were responsible for distributing and collecting consent forms and questionnaires to unit staff.

### Data analysis

#### Elicitation study

Conventional content analysis was employed with the narrative data once the full set of encounters was completed.^38^ Our approach involved journaling the data and coding, categorizing, and grouping of categories into major themes. Data related to the construction of the TPB questionnaires were separated out and specific commentary and recommendations were applied to the individual questionnaire items. Each category and theme were reviewed by all members of the team prior to including appropriate findings as questionnaire items. Additional findings were retained for informing future phases of the larger project.

#### Questionnaire

Data were entered into Microsoft Excel and exported to IBM-SPSS version 21 for analysis. Descriptive statistics (frequency and percentages) were used to profile study participants and pain management practices. Missing responses were included in the denominator for the calculation of percentages. Frequency and percentage were calculated for pain characteristics stratified by discipline and specialty. Responses to the questions that comprised each of the TPB subscales—Behavioral Intent, Attitudes, Subjective Norms, and Perceived Behavioral Control—were averaged to create subscale scores. Means and standard deviations were calculated for each of the subscales stratified by discipline and specialty. Cronbach’s alpha was used to test internal consistency of the TPB subscales; only the Behavioral Intent subscale had acceptable internal consistency (α = 0.81). Unpaired *t* test and analysis of variance were used to assess differences in behavioral intent across demographic and pain management characteristics. The general linear model was used to test the combined effect of attitudes, subjective norms, and perceived control on behavioral intent while controlling for discipline/specialty. Variables were manually removed from the multivariable model until only variables with *P* < 0.05 remained. Discipline/specialization was forced into the model.

## Results

### Objective 1: Elicitation study

Individual interviews and focus groups of three to six people were conducted with interdisciplinary staff at CHUK by three team members (GN, EV, RW). Nine encounters took place with a total of 32 health care professionals. Participants included surgical and anesthesiology residents; NAs and anesthesia technologists; staff nurses in the ICU, PACU, operating room, general surgery, and orthopedics units; one nurse manager; and one charge nurse.

Main discussion points were recorded along with verbatim exemplars by one team member (RW) during the course of each interview or focus group. It is important to note that there was variability in the responsiveness of participants to questions. Some participants were willing to engage and provided lengthy descriptions and commentary, whereas others responded only to direct questioning. The team made the decision not to record the discussion to facilitate open and honest dialogue. As a result, narrative transcription was limited to notes taken at the time and those added immediately after the interview by the team members.

### Discussion of questionnaire items and construction

There was some very interesting discussion in the focus groups about the subtleties in the distinction between verbs in the questionnaire items. In some cases, the participants suggested that some wording would not be easily understood; these items were modified to include words that were less alike and examples were used to provide clarity. Question 7, for example, includes the addition of “family/friends/colleagues” to provide clarity about this item because these individuals were identified as sources of social pressure in pain care practice. Consistent with the TPB approach to questionnaire development, item stems were constructed to include “people that are important to me (family/friends/colleagues).” Additionally, some of the TPB item Likert scales typically are presented as −3, –2, −1, 0, 1, 2, 3. Elicitation study participants were concerned that negative scoring would create a bias toward the positive end of the scale. As a result, all of the items were scored as one to seven in the final questionnaire.

### Additional discussion

Analysis of the additional narrative data resulted in the following main themes: knowledge and comfort with pain assessment; knowledge and comfort with pain treatment; medication and medication order availability; and fear of adverse effects of medications. The theme of fear of adverse effects was present in every interview and focus group discussion. This finding was not incorporated into the final questionnaire but instead was used by team members to inform educational content in a subsequent phase of the larger project. However, because of the consistency of this elicitation study finding in the narrative, an explanation has been included.

Specific questions were added to the questionnaire to address specific aspects of each theme. Table 1 provides the themes and corresponding additional questions included in the final questionnaire draft. Narrative data from the elicitation study regarding questionnaire item construction or comprehension were used to inform item modification.

**Table 1.** Additional questions.

Theme	Additional questions
Knowledge and comfort with pain assessment	20, 26, 29
Knowledge and comfort with pain treatment	21, 22, 25, 27
Medication and medication order availability	23, 24, 28

The final questionnaire contained 19 questions based on the TPB to assess the influence of attitude, subjective norms, and perceived behavioral control on the behavioral intent to treat postoperative pain. Responses were scored on a seven-point Likert scale. In all, nine questions were added about pain assessment and treatment knowledge, comfort, and practice and medication availability. Additionally, seven demographic, discipline and specialty, and location of work questions were included to provide a description of study participants (see Appendix).

### Fear of adverse effects

Fear of the adverse effects of strong analgesics can be best explained using an exemplar from one of the participants who explained the impact of this fear as “the nurses are scared to give morphine. They do not have the knowledge to deal with the complications—if the patient has trouble breathing. They don’t want to have to call the lung doctors. Patients complain but the nurses don’t give [spreads his hands wide and shrugs].” One nurse mentioned that he had experienced pressure from family members to get physicians to administer the pain medications rather than give the medication himself, reinforcing the notion that management of adverse effects was beyond the competency of nurses. The presence of a monitor mediated the fear of respiratory depression and hypotension. It was clear from the discussion that a physical device with oxygen saturation, blood pressure, and cardiac monitoring capabilities—present in the intensive care unit and recovery room—alleviated some of the nurses’ concern: “the nurses are not scared when the patient is on a monitor.”

### Objective 2: Questionnaire

One hundred and forty-one questionnaires were distributed and 136 were returned (96% response rate). Five participants held administrative roles and did not provide direct patient care and were therefore excluded, leaving 131 respondents for this analysis. Demographic and clinical characteristics of the study sample are presented in Table 2. Most study participants were either unit nurses (24%) or anesthesia technicians/nurses (28%). Forty-six percent indicated that they had training in acute pain as part of their health professional education program or in-service training. Pain management characteristics of the study sample stratified by hospital are reported in Table 3. Respondents from CHUK were significantly more likely to use the Numeric Rating Scale and/or a visual analogue scale compared to CHUB respondents (43% vs. 20%, respectively, *P* = 0.02), as well as the Faces Scale (61% vs. 35%, respectively, *P* = 0.01). At both sites, tramadol, morphine, and paracetamol were the most frequently used drugs to routinely treat acute pain, and morphine was the preferred drug for treating uncontrolled pain. Eighty-three percent of CHUK respondents were more likely to always or usually use a pain protocol compared to 57% of CHUB respondents (*P* < 0.01). When asked to report on limitations to treating acute pain, lack of availability of drugs was the most frequently cited factor (54% CHUK, 56% CHUB).

**Table 2.** Demographic and clinical characteristics of the sample.

Variable (sample *n*)	Description	*n* (%)
Location (131)	Kigali University Teaching Hospital	88 (67.2)
	Butare University Teaching Hospital	43 (32.8)
Language of questionnaire (131)	English	45 (34.4)
	French	86 (65.6)
Sex (130)	Male	58 (44.3)
	Female	72 (55.0)
Discipline/specialty (131)^a^	Anesthesiologist	2 (1.5)
	Surgeon	1 (0.8)
	Anesthesiology resident	2 (1.5)
	Surgery resident	11 (8.4)
	Nurse anesthetist/anesthesia technician	36 (27.5)
	PACU nurse	24 (18.4)
	ICU nurse	18 (13.7)
	Labor & delivery nurse	3 (2.3)
	Unit nurse	32 (24.4)
	General practitioner	2 (1.5)
Service (130)^b^	OB/GYN	62 (47.7)
	Orthopedics	88 (67.7)
	Pediatrics	53 (40.8)
	General surgery	88 (67.7)
	ICU	10 (7.6)
	Neurosurgery	6 (4.6)
	ENT/ophthalmology	4 (3.1)
Have pain training^c^ (129)	Yes	59 (45.7)
	No	70 (53.4)

PACU = postanesthetic care unit; ICU = intensive care unit; OB/GYN = obstetrics and gynecology; ENT = ear, nose, and throat.

^a^Participants were asked to select their primary role.

^b^Adds up to >100% because participants could select all that apply; for example, a nurse in PACU may have selected several services.

^c^Respondents were asked to describe their pain training. Responses included training acquired during their formal health professional education, single courses, and in-service activities.

**Table 3.** Pain management characteristics stratified by hospital.

		Kigali University Teaching Hospital Column *n* (%) (*n* = 88)	Butare University Teaching Hospital Column *n* (%) (*n* = 43)	Chi-square (*P* value)
Type of pain assessment scale used (130)^a^	Numeric Rating Scale and/or visual analogue scale	38 (43.2)	9 (20.9)	5.3 (0.02)
	Faces	53 (61.6)	15 (34.9)	6.5 (0.01)
Frequency of pain scale use (131)	Never	16 (18.2)	19 (44.2)	13.2 (<0.01)
	Rarely	10 (11.4)	6 (14.0)	
	Often	40 (45.5)	8 (18.6)	
	All the time	22 (25.0)	10 (23.3)	
Drugs used to treat pain (130)^a^	Tramadol	68 (78.2)	33 (76.7)	0.0 (0.86)
	Morphine	73 (83.9)	29 (67.4)	4.6 (0.03)
	Paracetamol	63 (72.4)	34 (79.1)	0.7 (0.41)
	Fentanyl	14 (16.1)	12 (27.9)	2.5 (0.11)
	Diclofenac	53 (60.9)	27 (62.8)	0.0 (0.84)
	Pethidine	53 (60.9)	4 (9.3)	31.1 (<0.01)
	Combination therapy	42 (47.7)	32 (74.4)	7.3 (<0.01)
Preferred drugs for uncontrolled pain (130)^a^	Tramadol	19 (21.8)	15 (34.9)	2.5 (0.14)
	Morphine	60 (69.0)	25 (58.1)	1.5 (0.22)
	Paracetamol	17 (19.5)	5 (11.6)	1.3 (0.26)
	Fentanyl	6 (6.9)	7 (16.3)	2.8 (0.09)^b^
	Diclofenac	16 (18.4)	4 (9.3)	1.8 (0.18)
	Pethidine	21 (24.1)	7 (16.3)	1.1 (0.31)
	Combination therapy	21 (23.9)	7 (16.3)	0.6 (0.44)
Frequency preferred drugs available (129)	Always/usually	8 (9.3)	9 (21.5)	10.6 (<0.01)
	Sometime	30 (34.9)	22 (52.4)	
	Rarely/never	48 (55.8)	11 (26.2)	
Frequency use pain protocol (129)	Always/usually	72 (82.7)	24 (57.1)	8.5 (<0.01)
	Sometime/rarely/never	15 (17.1)	18 (42.9)	
Provided follow-up pain care (130)	Yes	83 (95.4)	39 (90.7)	1.1 (0.29)
Provide follow-up pain care side effects (130)	Yes	80 (92.0)	39 (90.7)	0.1 (0.81)
Limitations to providing acute pain care (130)^b^	Availability of drugs	47 (54.0)	24 (55.8)	0.0 (0.85)
	Waiting for orders	41 (47.1)	14 (32.6)	2.5 (0.11)
	Fear of complications	16 (18.4)	9 (20.9)	0.1 (0.73)
	Lack of knowledge	2 (2.3)	1 (2.3)	0.0 (0.70)^b^
Preferred pain education delivery method (130)	In service	49 (56.3)	33 (76.7)	5.2 (0.02)
	Simulation	26 (29.9)	5 (11.6)	5.3 (0.02)
	Reading	11 (12.6)	6 (14.0)	0.0 (0.84)

^a^Adds up to >100% because participants could select all that apply.

^b^Fisher’s exact test.

Due to the limited sample size for some disciplines/specialties, they were categorized into four groups: physicians (*n* = 18, 14%), NAs (*n* = 36, 28%), PACU/ICU nurses (*n* = 42, 32%), and unit nurses (*n* = 35, 27%). There were statistically significant differences across discipline/specialty in the preferred drugs to treat uncontrolled pain and in the limitations to providing acute pain care (*P* < 0.01; Table 4). PACU/ICU nurses (46%) and unit nurses (29%) were more likely to prefer tramadol for uncontrolled pain compared to physicians (11%) and NAs (8%). The bivariate results for behavioral intent to treat postoperative pain are provided in Table 5. In general, the intent to treat postoperative pain was high, with mean scores for behavioral intent ranging from 5.7/7.0 (SD = 1.6) for unit nurses to 6.6 (SD = 0.4) for physicians (*P* = 0.01). There were no other statistically significant differences in mean behavioral intent scores across sample characteristics. Attitudes about assessing postoperative pain and perceived behavioral control (self-efficacy) were associated with the intent to treat postoperative pain, after controlling for specialty/discipline (Table 6). These two factors accounted for 26% of the variation in behavioral intent (*R*^2^ = 0.26).

**Table 4.** Pain characteristics stratified by discipline and specialty (*n* = 131).

		Staff physicians and residents (*n* = 18)	Nurse anesthetists/anesthesia technicians (*n* = 36)	PACU/ICU nurses (*n* = 42)	Unit nurses (*n* = 35)	
Variable (*n*)		*n* (column %)	*n* (column %)	*n* (column %)	*n* (column %)	Chi-square test (*P* value)
Frequency of formal pain scale use (131)	Often/always	9 (50)	18 (50)	26 (62)	27 (77)	6.6 (0.09)
	Never/rarely	9 (50)	18 (50)	16 (38)	8 (23)	
Frequency use pain protocol (129)	Always/usually	6 (35)	19 (53)	22 (54)	25 (71)	6.6 (0.09)
	Sometime/rarely/never	11 (65)	17 (47)	19 (47)	10 (29)	
Limitations to providing acute pain care (130)^a^	None	4 (27)	10 (35)	10 (26)	4 (13)	4.2 (0.25)
	Availability of drugs	13 (72)	23 (64)	20 (49)	15 (43)	6.0 (0.11)
	Waiting for orders	2 (11)	5 (14)	26 (63)	22 (63)	32.6 (<0.01)
	Fear of complications	5 (28)	3 (8.3)	10 (24)	7 (20)	4.3 (0.23)
	Lack of knowledge	0 (0.0)	1 (2.8)	1 (2.4)	1 (2.9)	0.5 (0.92)
Preferred pain education method (130)	In service	12 (67)	16 (44)	26 (63)	28 (80)	9.8 (0.02)
	Simulation	8 (44)	13 (36)	9 (22)	1 (2.9)	15.8 (<0.01)
	Reading	4 (22)	3 (8.3)	6 (15)	4 (11)	2.2 (0.53)
Have pain training (129)	Yes	11 (61)	13 (36)	10 (24)	25 (74)	21.2 (<0.01)
Preferred drugs for uncontrolled pain (130)	Tramadol	2 (11.1)	3 (8.3)	19 (46.3)	10 (28.6)	16.8 (<0.01)
	Morphine	13 (72.2)	20 (55.6)	31 (75.6)	21 (60.0)	4.3 (0.24)
	Paracetamol	2 (11.1)	4 (11.1)	4 (9.8)	12 (34.2)	10.3 (0.02)
	Diclofenac	0 (0.0)	3 (8.3)	4 (9.8)	13 (37.1)	18.4 (<0.01)
	Pethidine	3 (16.7)	10 (27.8)	6 (14.6)	9 (25.7)	2.6 (0.46)

PACU = postanesthetic care unit; ICU = intensive care unit.

^a^Adds up to >100% because participants could select all that apply.

**Table 5.** Behavioral intent to treat postoperative pain stratified by demographic, clinical, and pain management characteristics (bivariable comparisons).

Variable (*n*)		*n* (%)	Mean behavioral intent score (out of 7), mean (SD)	Test statistic	*P* value
Discipline and specialty (126)	Physicians	18	6.6 (0.4)	4.0	0.01
	Nurse anesthetist/anesthesia technician	35	6.5 (1.2)		
	PACU/ICU nurses	40	6.6 (0.9)		
	Unit nurses	33	5.7 (1.6)		
Care for the following types of patients					
OB/GYN patients (125)	Yes	60	6.4 (1.2)	0.3	0.59
	No	65	6.3 (1.2)		
Orthopedic patients (125)	Yes	84	6.4 (1.3)	0.4	0.53
	No	41	6.2 (1.1)		
Pediatric patients (125)	Yes	50	6.6 (1.0)	3.2	0.08
	No	75	6.2 (1.3)		
General surgery patients (125)	Yes	86	6.3 (1.2)	0.0	0.97
	No	39	6.4 (1.3)		
Have pain training (124)	Yes	56	6.2 (1.3)	1.9	0.18
	No	68	6.5 (1.1)		
Frequency of pain scale use (126)	Never/rarely	50	6.4 (1.0)	0.0	0.94
	Often/all the time	76	6.3 (1.3)		
Frequency use pain protocol (124)	Sometime/rarely/never	54	6.1 (1.5)	2.5	0.11
	Always/usually	70	6.5 (0.9)		
Limitations to providing acute pain care					
None (109)	Yes	82	6.3 (1.2)	0.8	0.94
	No	27	6.4 (1.1)		
Availability of drugs (125)	Yes	69	6.3 (1.2)	0.4	0.73
	No	56	6.4 (1.2)		
Waiting for orders (125)	Yes	52	6.2 (1.4)	1.4	0.16
	No	73	6.5 (1.1)		
Fear of complications (125)	Yes	25	6.6 (0.6)	1.2	0.22
	No	100	6.3 (1.3)		
Lack of knowledge (125)	Yes	3	6.4 (0.7)	0.1	0.89
	No	122	6.3 (1.2)		

PACU = postanesthetic care unit; ICU = intensive care unit; OB/GYN = obstetrics and gynecology.

**Table 6.** Relationship between theory of planned behavior–independent factors and behavioral intent to treat postoperative pain while controlling for discipline/specialization.

Variable (n)	Mean (SD)	Bivariable Pearson correlation (*P* value)	Multivariable correlation with behavioral intent^a^ F statistic (*P* value)
Attitudes assessment (127)	6.6 (0.7)	0.41 (<0.01)	16.5 (<0.01)
Attitudes about treatment (127)	6.7 (0.7)	0.26 (<0.01)	Removed
Subjective norms (123)	5.2 (1.1)	0.16 (0.09)	Removed
Perceived behavioral control–self-efficacy (124)	5.1 (1.5)	0.30 (<0.01)	9.1 (<0.01)
Perceived behavioral control–controllability (125)	4.2 (1.5)	0.09 (0.34)	Removed

^a^*R*^2^ = 0.26, adjusted for specialty/discipline (F = 1.8, *P* = 0.15). Order in which variables were removed from multivariable analysis: (1) attitudes about treatment, (2) perceived behavioral control–controllability, (3) subjective norms.

## Discussion

Several systemic and knowledge barriers to assessing and treating postoperative pain exist in Rwanda, including limited use of evidence-based approaches to assessing and treating pain. However, the most frequently cited barrier to treating postoperative pain was system related; that is, inconsistent or lack of availability of appropriate medication. The overall intent to treat postoperative pain was high, with attitudes about assessing postoperative pain and perceived behavioral control (self-efficacy) accounting for 26% of the variation in behavioral intent.

Consistent with other reports in the literature, systemic barriers have a deleterious impact on the treatment of postoperative pain in the Rwandan context. For example, most participants cited morphine as the drug of choice for postoperative pain, but there are barriers to supplying opioids to public hospitals and clinics in African countries, including difficulty sourcing and high prices.^39^ Per the World Health Organization, low-resource countries accounted for 6% of opioid consumption compared to 79% for six developed nations.^39^ Thus, it is important to consider the context—for example, availability of morphine and other opioids—when assessing the appropriate use of analgesia and best-evidence protocols in general in low-resource settings.

Our findings support the earlier study conducted in Rwanda by Johnson et al., where the lack of ongoing continuous education and fear of making incorrect decisions about the choice of medications were identified as major factors to evidence-based practice.^22^ In most African countries, it has been shown that despite education during formal academic training, once in practice there is often no continuing education, which leads to loss of basic knowledge.^40^ In the current study, 46% of all respondents and 61% of medical personnel reported having acute pain training. Given that pain training is routinely provided in anesthesiology and nursing training programs, some participants may have interpreted this question to mean pain education in addition to what they received during formal training. The proportion of nurses reporting acute pain training in this study is lower than what was reported in emergency department nurses in central Africa (75%); however, in that study, participants were specifically asked about pain education during their formal educational programs.^19^ Another knowledge-related barrier was fear of administering opioids and not having the ability to recognize and deal with adverse effects. Nurses reported feeling that they worked in isolation because they believed that they were unable to administer opioids without a physician being present, preferably the anesthesia staff, even if there had been an order to do so. These findings are consistent with the report by Rampanjato et al. where 68% of emergency department nurses reported being afraid to administer morphine.^19^ The findings of this study underline the importance of establishing continuing education, quality improvement, and sustainability programs in pain management for nurses and other health professionals.

This study was the first to review perioperative pain management practice in Rwanda, using self-reported information and an established framework, the TPB,^35,36^ to identify potential barriers to providing pain medication. The questionnaires was developed and reviewed for language and comprehension in collaboration with health care personnel in Rwanda. Questionnaire distribution included a Rwandan team member who reviewed each question on the questionnaire with the key stakeholder on each unit, provided additional information as necessary, and answered questions in any of the three languages. The close to 100% participation rate can be attributed to the integrated approach to questionnaire development and data collection. Data collection occurred in two of the three academic referral hospitals in Rwanda, making the results generalizable to tertiary care in Rwanda and possibly other low-resource settings but not necessarily to other levels of health care provision in Rwanda. Weaknesses of the study include the potential limitations of the TPB in the Rwandan context, primarily due to the difficulty that some participants may have had in distinguishing between some of the concepts on the questionnaire (e.g., differentiating between response such as good vs. bad and harmful vs. beneficial). The results of the study are based on self-report, which is appropriate given that participants were asked to report on their perceptions and their preferences. However, the interpretation of some questions may have varied between participants (e.g., how they defined acute pain training), making it difficult to assess the true state of pain education.

The results of this study can be used to develop protocols and guidelines to improve the quality of postoperative pain management in Rwanda. They could also be used as a baseline for future studies to examine the impact of developing and implementing context-sensitive postoperative pain protocols in Rwanda. Adapting protocols and guidelines to the local context of other low-resource countries may also facilitate best practices in settings where access to medication may be limited.

Future studies should also examine the impact of patient education on the ability to adequately assess pain. Chaibou et al. found that illiteracy and lack of medical knowledge impacted the ability of patients to comprehend the use of validated pain tools like the NRS or visual analogue scale.^5^ In addition, patients’ fears of addiction to opioids and side effects may impair their acceptance of these medications, even when available.^14–16^ The inclusion of patient education toward creating an expectation of appropriate pain care has the potential to impact practice and pain-related outcomes. The overall findings of this study support the need for advocacy related to the establishment of an institutional culture and expectation for appropriate pain care through the creation of evidence-informed practices and guidelines that are relevant to the Rwandan context and administrative-level support for improving resources (e.g., increasing the availability of medications and treatments).

## Appendix

### Section 1

Each question in this section **refers to treating acute pain**. (Please circle the number on each line that applies. For example in question 1, the 1 means “easy,” 7 means “difficult,” and 4 means “neutral [i.e., not easy or difficult]”)

**Section 2**
